# A mannitol-modified emodin nano-drug restores the intestinal barrier function and alleviates inflammation in a mouse model of DSS-induced ulcerative colitis

**DOI:** 10.1186/s13020-023-00801-0

**Published:** 2023-08-11

**Authors:** Yin-Yue Xu, Min Zhu, Jiang Wu, Long-Biao Luo, Si-jing Dong, Meng-Gai Zhang, Xue Liu, Ke Wang, Hua Luo, Wang-Hui Jing, Lin Wang, Si-Cen Wang

**Affiliations:** 1 School of Pharmacy, Health Science Center, Xi’an Jiaotong University, Xi’an, 710061 China; 2 Shaanxi Engineering Research Center of Cardiovascular Drugs Screening and Analysis, Xi’an, 710061 China; 3 College of Chemistry and Pharmacy, Northwest A&F University, Yangling, 712100 Shaanxi China; 4 State Key Laboratory of Quality Research in Chinese Medicine, Macau Centre for Research and Development in Chinese Medicine, Institute of Chinese Medical Sciences, University of Macau, Macao, China; 5 State Key Laboratory of Molecular Engineering of Polymers (Fudan University), Shanghai, 200438 China

**Keywords:** Ulcerative colitis, Emodin, Nano drug delivery system, Intestinal barrier

## Abstract

**Background:**

Ulcerative colitis (UC) is an inflammatory disease of the colon that is characterized by mucosal ulcers. Given its increasing prevalence worldwide, it is imperative to develop safe and effective drugs for treating UC. Emodin, a natural anthraquinone derivative present in various medicinal herbs, has demonstrated therapeutic effects against UC. However, low bioavailability due to poor water solubility limits its clinical applications.

**Methods:**

Emodin-borate nanoparticles (EmB) were synthesized to improve drug solubility, and they modified with oligomeric mannitol into microgels (EmB-MO) for targeted delivery to intestinal macrophages that express mannose receptors. UC was induced in a mouse model using dextran sulfate sodium (DSS), and different drug formulations were administered to the mice via drinking water. The levels of inflammation-related factors in the colon tissues and fecal matter were measured using enzyme-linked immunosorbent assay. Intestinal permeability was evaluated using fluorescein isothiocyanate dextran. HE staining, in vivo imaging, real-time PCR, and western blotting were performed to assess intestinal barrier dysfunction.

**Results:**

Both EmB and EmB-MO markedly alleviated the symptoms of UC, including body weight loss, stool inconsistency, and bloody stools and restored the levels of pro- and anti-inflammatory cytokines. However, the therapeutic effects of EmB-MO on the macroscopic and immunological indices were stronger than those of EmB and similar to those of 5-aminosalicylic acid. Furthermore, EmB-MO selectively accumulated in the inflamed colon epithelium and restored the levels of the gut barrier proteins such as ZO-1 and Occludin.

**Conclusions:**

EmB-MO encapsulation significantly improved water solubility, which translated to greater therapeutic effects on the immune balance and gut barrier function in mice with DSS-induced UC. Our findings provide novel insights into developing emodin-derived drugs for the management of UC.

## Introduction

Ulcerative colitis (UC) is a chronic inflammatory bowel disease (IBD) that primarily occurs in the colon and manifests as mucosal ulcers and progressive destruction of the intestinal barrier function [1]. It has become a global health challenge due to the rapidly increasing incidence rates and the tremendous burden on health care systems [2]. Recent studies have shown that the younger population is more susceptible to UC [2]. In addition, UC patients are more prone to anxiety and depression [3], and the risk of neoplastic transformation of ulcerative lesions in UC patients with a disease course longer than 10 years is continually increasing [4]. Various factors have been implicated in the pathogenesis of UC, including disordered immune responses, intestinal barrier damage, genetic susceptibility, intestinal dysbiosis, and environmental stresses; however, the exact mechanisms are unknown [5–7]. Currently, UC is treated with sulfasalazine, glucocorticoids, immunosuppressants, and other biological agents, all of which have some disadvantages, such as limited efficacy, obvious side effects, or high costs [8, 9]. Therefore, it is of utmost importance to develop safe and effective drugs to treat UC.

Numerous studies have shown that Chinese herbal medicine has promising prospects in the treatment of UC owing to its potent anti-inflammatory pharmacological effects and intestinal mucosal barrier protective actions [10–13]. Emodin is a natural anthraquinone derivative that is present in various medicinal herbs, including *Rheum palmatum* L., *Polygonum cuspidatum* Sieb. et Zucc., and *Polygonum multiflorum* Thunb. It has well-documented anti-inflammatory, antioxidant, antimicrobial, anti-cancer, hepatoprotective [14–17], and other pharmacological properties [18, 19]. A recent study showed that Em could alleviate UC symptoms by regulating the flagellin-TLR5 dependent pathway [20–22]. However, the low water solubility and rapid metabolism of Em in the duodenum and jejunum result in a low bioavailability of primary oral formulations, which eventually limits its therapeutic efficacy [23]. In addition, pharmacokinetic analysis in mice with DSS-induced UC has shown that the systemic levels of Em increase significantly following oral administration, which might increase the risk of toxic adverse effects [24]. Therefore, it is essential to develop a carrier for the in situ delivery of Em to overcome the above shortcomings and achieve better therapeutic effects [25].

Oral colon targeted drug delivery system (OCDDS) can deliver drugs directly to colon lesions [26], which not only optimizes the drug concentration at the target site and enhances its therapeutic effects but also reduces the drug dose, improves patient compliance [27, 28], and minimizes damage to healthy tissues [29]. Intestinal macrophages have been reported as a key factor in the pathogenesis of UC by exerting a proinflammatory role [30, 31]. Macrophages are the most abundant immune cells in the intestinal tract [32] and express high levels of mannose receptors [33]. Accumulating evidence has demonstrated that under inflammatory conditions, macrophages secrete proinflammatory cytokines and significantly accelerate the pathological progress of IBD [34–37]. Overexpressed mannose receptors on the surface of intestinal macrophages encouraged the development of mannosylated OCDDS that could target the inflammatory colon lesions of macrophage aggregation. Therefore, as a common ligand of mannose receptor, oligomeric mannitol was utilized to modify Em, which could then selectively bind to intestinal macrophages. As macrophages are primary effectors of inflammation, the modification of anti-colitis drugs with mannitol can help achieve targeted delivery to the inflamed colonic lesions [38].

The poor water solubility of Em is a critical problem that affects its therapeutic effects. Given the poor water solubility of Em, we synthesized water-soluble Em-borate nanoparticles (EmB) via the solvent exchange method and borate esterification reaction [39]. Despite the distinctive biological activities, the adverse effects of Em, including hepatorenal toxicity and cytotoxicity, are of wide concern worldwide [40]. Thus, with the aim of lowering the toxicity and enhancing the therapeutic effects, we modified EmB with oligomeric mannitol to obtain a microgel preparation (EmB-MO) that can target inflammatory colon lesions. The therapeutic potential of EmB and EmB-MO were tested in a mouse model of dextran sulfate sodium (DSS)-induced UC in terms of disease activity index (DAI), inflammatory indices, structural changes in the colon, and intestinal barrier function.

## Materials and methods

### Materials

Emodin (Em, Mw ca. 270.24) was purchased from Sigma-Aldrich. Borax (Mw ca. 381.37, AR grade) was purchased from Tianjin Bodi Chemical Co. Ltd. Oligomeric mannitol (MGS, Mw ca. 342.3) was obtained from Xi'an Lavia Biotechnology Co. Ltd. Cy7-labeled EmB-MO was obtained from Xi'an Ruixi Biotechnology Co. Ltd. Other chemicals and solvents were of analytical reagent grade and used according to the standards. All experiments were performed using deionized water.

### Animals

A total of 48 male C57BL/6 mice (SPF grade, 6–8 weeks old, body weight 18–20 g) were obtained from the Experimental Animal Center of Xi'an Jiaotong University. The experimental procedures were performed as per the "Guidelines for the Care and Use of Laboratory Animals" and approved by the ethics committee (approval number: XJTU2019-679). The mice were housed at ambient temperature and relative humidity (55% ± 5%) under normal circadian rhythm, with food and water provided ad libitum.

### Preparation of EmB nanoparticles

EmB was fabricated as described in our previous study [39]. Briefly, 80.5 mg Em was dissolved in 10 mL DMSO, and 2.25 mL of this solution was added dropwise to 4 mL aqueous borax (50 mM) with intense stirring. The mixture was allowed to react at room temperature in the dark for 12 h, and a homogenous hydrosol of EmB (2.9 mg/mL) was obtained. 

### Preparation of EmB-MO microgels

Briefly, 3 mL of the EmB hydrosol was added dropwise into 3 mL aqueous mannitol oligomer solution and constantly stirred for > 4 h at room temperature. The resulting EmB-MO microgels were lyophilized.

### Characterization of EmB-MO microgels

The digital images of the microgel samples were captured using Nikon D3100 camera. The infrared spectral images were obtained on the transform infrared spectrometer (Nicolet IS-10). The diluted EmB hydrosol and EmB-MO (0.29 mg/mL) microgel preparations were poured on silicon wafers and observed using a scanning electron microscope (SEM, HITACHI S-4800). The hydrodynamic diameter and zeta potential of the samples were measured at room temperature using Malvern ZS90 Zetasizer.

### Construction of DSS-induced UC model

The animals were randomly divided into the healthy control, UC model, Em (8 mg/kg/d), EmB-L (3 mg/kg/d), EmB-H (6 mg/kg/d), EmB-MO-L (3 mg/kg/d), EmB-MO-H (6 mg/kg/d), and 5-ASA (100 mg/kg/d) groups (n = 6 mice per group). UC was induced by supplementing drinking water with 3% DSS for 7 consecutive days. At the same time, water (equal quantity) was administered to the healthy control and UC mice via oral gavage. During the dosing period, disease activity index (DAI), body weight, stool consistency, and stool blood content were monitored daily. On day 8, the animals were sacrificed and dissected, and their spleen and colon tissues were weighed. The colon tissues were dissected and stored at − 80 °C for further analysis.

### Hematoxylin–eosin (H&E) staining

The colon tissues were removed for histological analysis. In addition, the heart, liver, spleen, lung, and kidney of mice in the control and EmB-MO-H groups were isolated for toxicological assessment. The tissues were fixed in 10% formalin for 48 h, dehydrated through an alcohol gradient, clarified in xylene, embedded in paraffin, and then cut into 3–4 µm thick sections. The sections were deparaffinization with xylene and then stained with H&E as per standard protocols. The slides were imaged using Panoramic DESK (P-MIDI, P250), and the size of the ulcers, infiltration of inflammatory cells, and degree of tissue damage were evaluated. The pathological score was assessed as previously described [41]. In brief, the total score was calculated from the inflammatory cell infiltration score (0–4), mucosal thickening score (0–4), goblet cell depletion score (0–4), structure destruction score (0 or 3–4), and crypt loss score (0 or 3–4). The maximum score was 20.

### Myeloperoxidase (MPO) assay

MPO activity in the colon tissues was detected using a specific kit according to the manufacturer’s instructions. The colon tissues were homogenized in PBS and centrifuged, and the supernatant was collected for the assay.

### Enzyme-linked immunosorbent assay (ELISA)

The fecal samples and colon tissues were prepared as previously described[12]. Lipoprotein-2 (Lcn-2) levels in the feces and IL-6, IL-1*β*, TNF-*α* and IL-10 levels in colon homogenates were measured using the respective ELISA kits as per the manufacturer’s instructions.

### Intestinal permeability assay

Fluorescein isothiocyanate dextran (FITC-dextran) was used to measure intestinal permeability as described previously [12]. Mice were deprived of food and water for 4 h and then orally administered with 4 kDa FITC-dextran (60 mg/100 g). After 5 h, blood was collected from the retro-orbital region and fluorescence intensity was measured at 525 nm. The FITC-dextran concentration in the serum was then calculated using a standard curve.

### Western blotting

Total protein was extracted from colon tissues using RIPA lysis buffer and quantified using the BCA method. Then, equal amounts of protein per sample were separated using 10% SDS-PAGE and transferred to polyvinylidene fluoride membranes. After blocking with 5% fat-free milk in Tris-buffered saline + Tween 20 (TBST), the membranes were incubated overnight with primary antibodies targeting ZO-1 (1:1000, Cat #ab276131; Abcam), Occludin (1:2000, Cat #ab216327; Abcam), and GAPDH (1:5000, Cat #ab8245; Abcam) at 4 °C. The following day, the membranes were incubated with secondary antibodies and developed using Chemistar High-sig ECL Western Blotting Substrate. The density of the bands was quantified using Image-Pro Plus 6.0 software.

### Real-time PCR

Total RNA was extracted from colon tissues with TRIzol (Invitrogen) and reverse-transcribed to cDNA using the Maxima First Strand cDNA Synthesis Kit (Fermentas) according to the manufacturer’s instructions. RT-PCR was performed on the CFX96 Touch Real-Time PCR detector (Bio-Rad, USA). Relative gene expression was measured and normalized to that of GADPH using the 2^−ΔΔCt^ method. The primers were as follows: (Claudin 1: 5'-TGGGGACAACATCGTGACTG-3' and 5'-CCCCAGCAGGATGCCAATTA-3'; Claudin 4: 5'-TGGAACCCTTCCGTTGATTA-3' and 5'-CACTGGGCTGCTTCTAGGTC-3'; ZO-1: 5'-TTTTTGACAGGGGGAGTGG-3' and 5'-TGCTGCAGAGGTCAAAGTTCAAG-3'; Occludin: 5'-ATGTCCGGCCGATGCTCTC-3' and 5'-TTTGGCTGCTCTTGGGTCTGTAT-3'; and GAPDH: 5'-AGGTCGGTGTGAACGGATTTG-3' and 5'-GGGGTCGTTGATGGCAACA-3').

### In vivo imaging

The biodistribution of the drugs was analyzed using an in vivo imaging system (IVIS). The mice were fasted for 12 h with water provided ad libitum. After weighing the mice, cy7-labeled Em, cy7-labeled EmB, and cy7-labeled EmB-MO microgels were administered via oral gavage. Twelve hours later, the mice were euthanized and the major organs (stomach, colon, and small intestine) were removed. The fluorescence signals in the different tissues were detected using the IVIS SPECTRUM animal imaging system with excitation wavelength (E_*x*_) and emission wavelength (E_*m*_) of 749 nm and 776 nm, respectively. Living Image software was used to analyze the images.

### Statistical analysis

Statistical analysis was performed using GraphPad Prism 8.0 (GraphPad, San Diego, CA). All quantitative data are presented as the mean ± standard error of the mean. Differences were compared using one-way or two-way analysis of variance, followed by Tukey’s multiple comparison test. *P* < 0.05 was considered statistically significant.

## Results and discussion

### Preparation and characterization of EmB and EmB-MO microgels

**Fig. 1:**
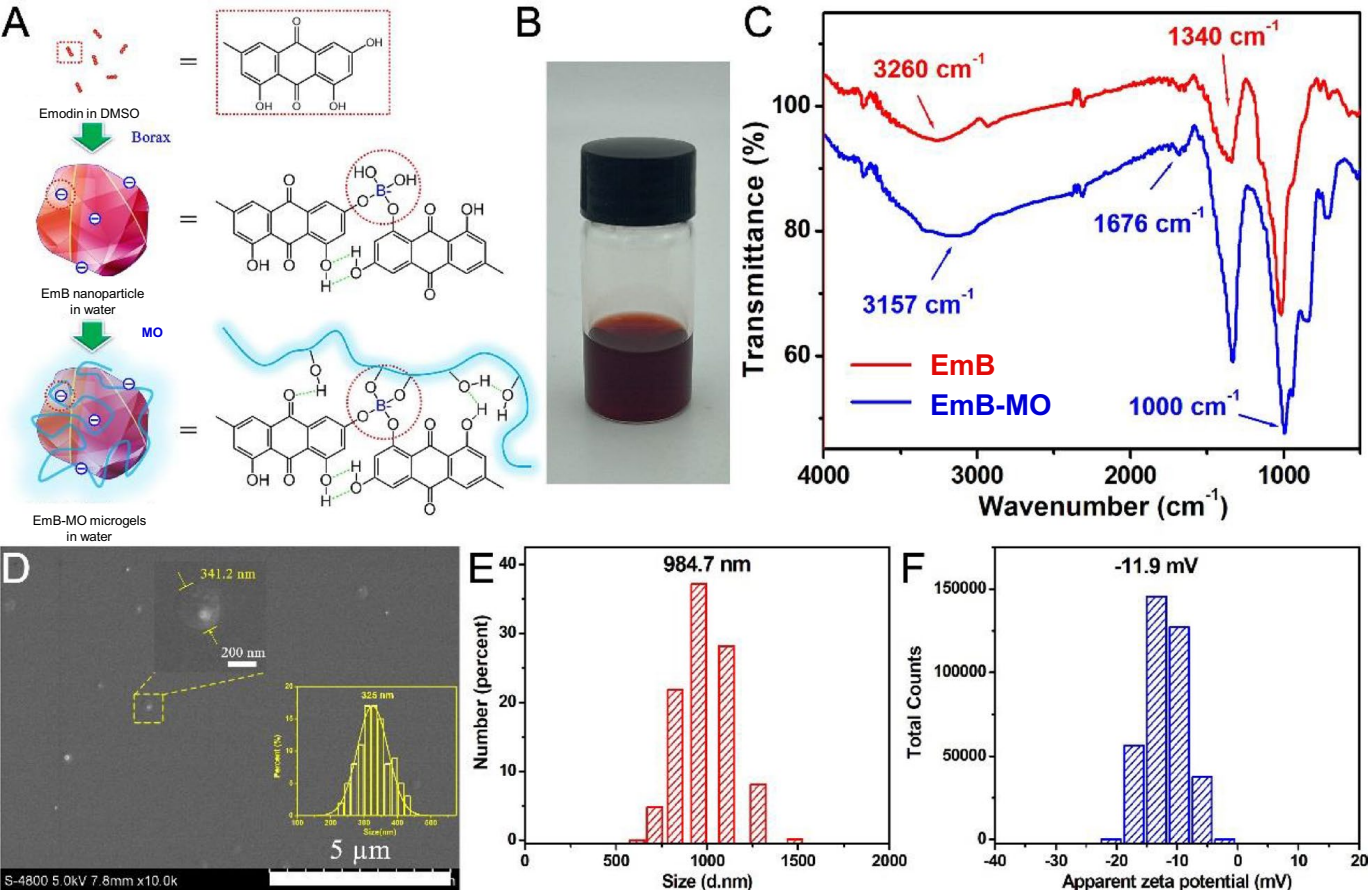
Preparation and characterization of EmB and EmB-MO microgels. **A** Synthesis steps of EmB and EmB-MO. **B** Representative images of EmB-MO microgels. **C** FTIR spectra of EmB and EmB-MO microgels. **D** Representative SEM image of EmB-MO microgels. Particle size and size distribution of EmB-MO microgels in borax solution. **F** Zeta potential of EmB-MO microgels in borax solution

Despite its excellent antibacterial and anti-inflammatory effects [42], the clinical application of Em is limited due to low bioavailability and poor water solubility (0.25 μg/mL) [23]. In a previous study, we synthesized water-soluble EmB that were formed through borate ester bonds on the surface of Em. The solubility of EmB increased significantly to 2.94 mg/mL [39]. Therefore, 2.9 mg/mL EmB were crosslinked with mannitol oligomers via hydrogen bonding to generate EmB-MO microgels. The mannitol oligomers can achieve targeted delivery of the drugs to the macrophages accumulating in the inflamed lesions on the intestinal wall by binding with their mannose receptors [33]. The synthesis steps of EmB and EmB-MO microgels are shown in Fig. 1A, and the representative image of the stable microgel preparation is shown in Fig. 1B.

EmB and EmB-MO were characterized using FTIR, SEM, DLS, and zeta potential measurements (Fig. 1C–F). As shown in the FTIR spectra for EmB (curve a) and EmB-MO (curve b) in Fig. 1C, the peak corresponding to the phenolic -OH of Em appeared at 3392 cm^−1^, which was also reported in our previous study [39]. However, the peak in EmB shifted to 3446 cm^−1^ owing to the formation of hydrogen bonds between the -OH groups of Em and borate ions [39], and the peak at 1676 cm^−1^ corresponded to the C = O stretching frequency of Em [43]. In the spectrum of EmB-MO microgels, we clearly observed a significant shift of the peak at 3446 cm^−1^ corresponding to the -OH groups of EmB to 3157 cm^−1^, which can be attributed to the hydrogen bonding between mannitol oligomers and EmB. Furthermore, the peak at 1676 cm^−1^ corresponding to the C = O stretching frequency of Em was evident in the spectrum of the EmB-MO microgels. These results indicated that EmB was combined with mannitol oligomers via hydrogen bonding. The morphology of the nanoparticles was examined using SEM. As shown in Fig. 1D, the EmB-MO microgel particles were round with an average diameter of approximately 322.6 ± 43.51 nm. In addition, the surface of EmB was covered with mannitol oligomers, indicating the formation of microgels in borax solution. The size distribution and zeta potential of EmB-MO microgels in solution were measured using DLS. As shown in Fig. 1E, F the average hydrodynamic diameter of the EmB-MO microgels was 984.7 ± 144.6 nm. The size of the microgels measured via DLS was larger than that observed using SEM, which can be explained by the expansion of MO chains in water and their contraction in the absence of water. The average zeta potential of EmB-MO microgels was -11.90 ± 3.26 mV, which further proved that the negatively charged EmB particles were successfully crosslinked with MO.

### EmB and EmB-MO alleviated the symptoms of DSS-induced UC

**Fig. 2:**
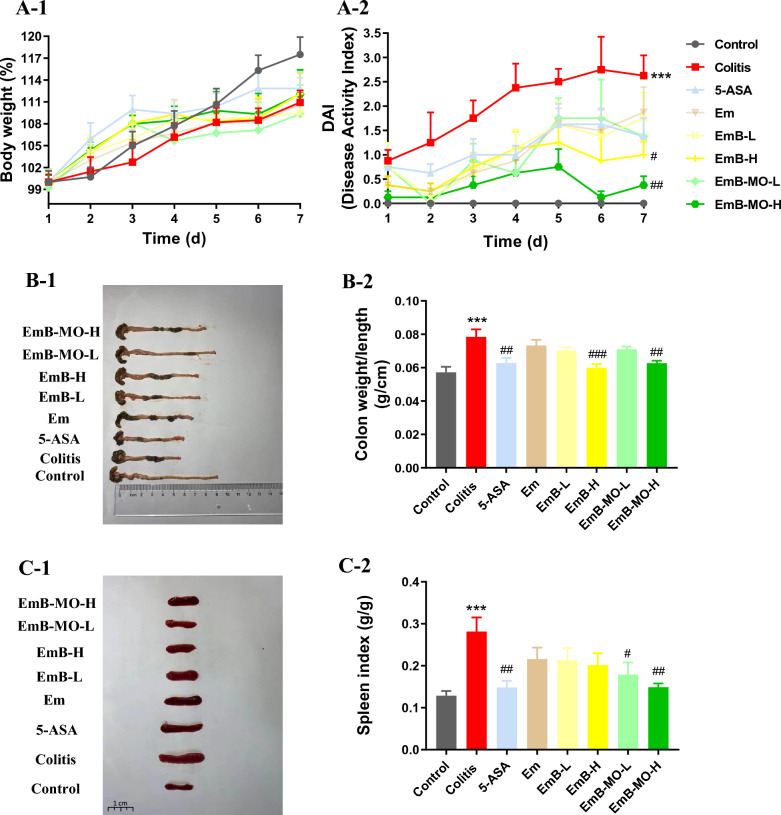
EmB-MO improved the pathological symptoms in mice with DSS-induced ulcerative colitis. **A** Daily changes in body weight (**A-1**) and disease activity index (DAI) (**A-2**) in the indicated group. **B** Macroscopic observation of colon (**B-1**) and the ratio of colon weight to length (**B-2**). **C** Macroscopic observation of spleen (**C-1**) and the ratio of spleen to body weight (**C-2**). Data represent the mean ± standard error of the mean (n ≥ 6). ^***^P < 0.001 vs. control group, ^#^*P* < 0.05, ^##^*P* < 0.01, ^###^*P* < 0.001 vs. ulcerative colitis group. 5-ASA: 5-aminosalicylic acid, 100 mg/kg/d; Em: Emodin, 8 mg/kg/d; EmB-L: Emodin-borate nanoparticles, 3 mg/kg/d; EmB-H: 6 mg/kg/d; EmB-MO-L: EmB modified with oligomeric mannitol into microgels, 3 mg/kg/d; and EmB-MO-H: 6 mg/kg/d

**Fig. 3:**
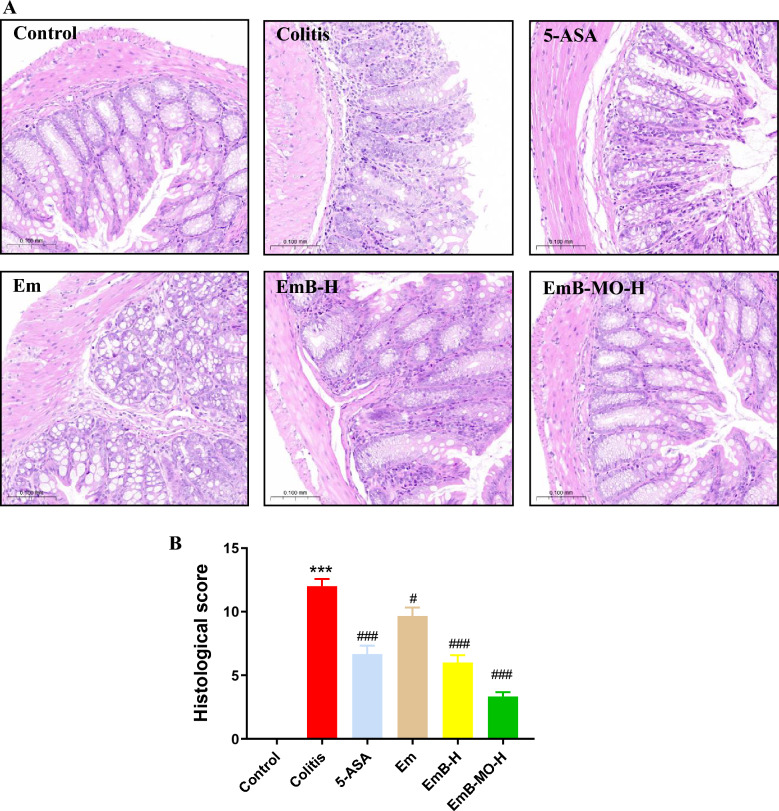
EmB-MO restored the integrity of colon tissue in mice with DSS-induced ulcerative colitis. **A** Representative images of hematoxylin and eosin (H&E)-stained colon tissues. Scale bar = 100 µm. **B** Histological scores of colon tissues in each group. Data represent the mean ± standard error of the mean (n = 3). ^***^P < 0.001 vs. control group, ^#^*P* < 0.05, ^###^*P* < 0.001 vs. ulcerative colitis group. 5-ASA: 5-aminosalicylic acid, 100 mg/kg/d; Em: Emodin, 8 mg/kg/d; EmB-L: Emodin-borate nanoparticles, 3 mg/kg/d; EmB-H: 6 mg/kg/d; EmB-MO-L: EmB modified with oligomeric mannitol into microgels, 3 mg/kg/d; and EmB-MO-H: 6 mg/kg/d

DSS-induced UC simulates the typical symptoms of IBD, including bloody stools, diarrhea, and weight loss [44]. The mice treated with 3% DSS showed a slower increase in body weight than those in the control group, which was accompanied by inconsistent and bloody stools. Moreover, DAI gradually improved after DSS induction, indicating the successful induction of UC. By contrast, DAI in mice treated with EmB and EmB-MO was markedly lower (Fig. 2A), particularly in the EmB-MO group. UC is characterized by significant colon shortening and splenomegaly [45]. Consistently, we recorded greater colon weight/length ratio and enlarged spleen in the UC group, which was reversed by high doses of EmB-MO (Fig. 2B, C). Finally, the pathological examination of the colon sections revealed inflammatory cell infiltration, crypt destruction, and mucosal damage after DSS-induced UC, all of which improved remarkably after EmB-MO administration (Fig. 3A, B). Taken together, EmB and EmB-MO alleviated the pathological changes associated with DSS-induced UC, and EmB-MO exhibited better therapeutic effects than EmB.

### EmB and EmB-MO alleviated colonic inflammation in DSS-treated UC mice

**Fig. 4:**
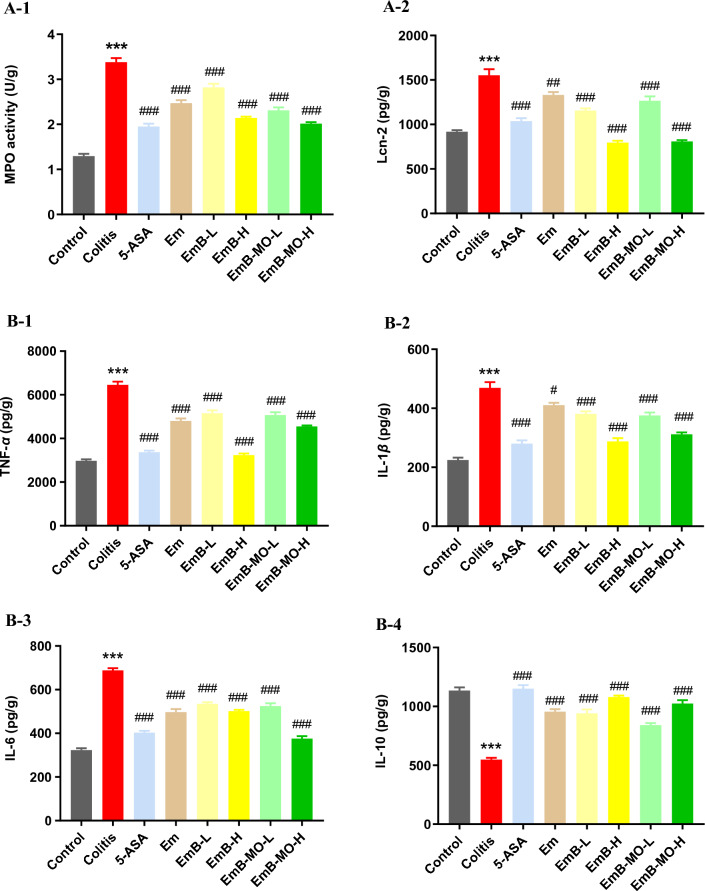
EmB-MO attenuated the inflammatory response in mice with DSS-induced ulcerative colitis. **A** MPO activity in colon tissues and Lcn-2 activity in feces. **B** Expression levels of TNF-*α* (B-1), IL-1*β* (**B-2**), IL-6 (**B-3**), and IL-10 (**B-4**) in colon tissues. Data represent the mean ± standard error of the mea (n = 3). ^***^P < 0.001 vs. control group, ^#^*P* < 0.05, ^##^*P* < 0.01, ^###^*P* < 0.001 vs. ulcerative colitis group. 5-ASA: 5-aminosalicylic acid, 100 mg/kg/d; Em: Emodin, 8 mg/kg/d; EmB-L: Emodin-borate nanoparticles, 3 mg/kg/d; EmB-H: 6 mg/kg/d; EmB-MO-L: EmB modified with oligomeric mannitol into microgels, 3 mg/kg/d; and EmB-MO-H: 6 mg/kg/d

Neutrophil infiltration is a parameter of the severity of UC, and activated neutrophils release large amounts of MPO during inflammation. In addition, the presence of Lcn-2 or neutrophil gelatinase-associated lipocalin in the feces is a biomarker of intestinal inflammation [46]. Consistent with this, MPO activity in the colon and Lcn-2 levels in feces were elevated in the UC model. As shown in Fig. 4A, Em, EmB, and EmB-MO significantly inhibited colonic MPO activity and reduced fecal Lcn-2 levels in the UC mice. In addition, DSS-induced UC was associated with the increased levels of several pro-inflammatory cytokines, including TNF-*α*, IL-6 and IL-1*β*, and a concomitant decrease in the anti-inflammatory cytokine IL-10 in colon tissues. As expected, Em, EmB, and EmB-MO treatment restored the levels of all inflammation-related cytokines (Fig. 4B). However, despite its significant anti-inflammatory effects, Em treatment did not lead to the remission of UC symptoms such as weight loss, bloody stools, and splenomegaly. Therefore, we surmised that the therapeutic effects of EmB and EmB-MO are dependent on additional mechanisms, such as restoration of the intestinal barrier function.

### EmB-MO restored the intestinal barrier function in mice with DSS-induced UC

**Fig. 5:**
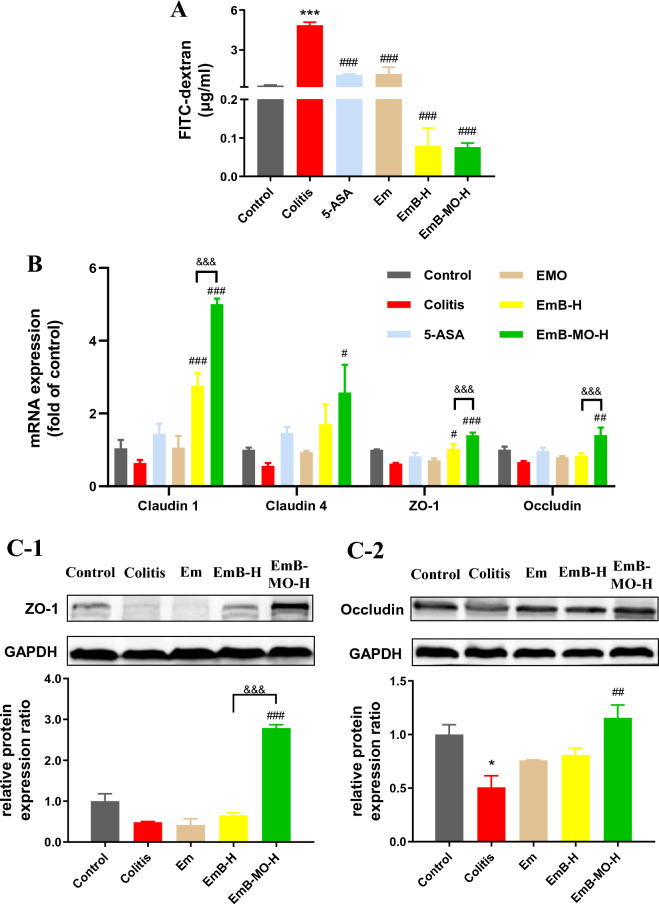
EmB-MO restored the intestinal barrier function in mice with DSS-induced ulcerative colitis. **A** Intestinal permeability of FITC-dextran in the indicated different groups. **B** Claudin 1, Claudin 4, ZO-1, and Occludin mRNA levels in colon tissues. **C** Representative immunoblots showing expression levels of ZO-1 (**C-1**) and Occludin (**C-2**). The relative protein expression was normalized to GAPDH. Data represent the mean ± standard error of the mean (n = 3). ^*^P < 0.05, ^***^P < 0.001 vs. control group, ^#^*P* < 0.05, ^##^*P* < 0.01, ^###^*P* < 0.001 vs. ulcerative colitis group, ^&&^*P* < 0.01, ^&&&^*P* < 0.001 vs. EmB-MO-H group. 5-ASA: 5-aminosalicylic acid, 100 mg/kg/d; Em: Emodin, 8 mg/kg/d; EmB-H: Emodin-borate nanoparticles, 6 mg/kg/d; and EmB-MO-H: EmB modified with oligomeric mannitol into microgels, 6 mg/kg/d

The remission of the histopathological signs of UC correlates with lower risks of hospitalization, colectomy, and colorectal cancer [47, 48]. As mucosal healing has been considered a key endpoint in UC therapy, we evaluated the effects of Em, EmB, and EmB-MO on the permeability of the intestinal epithelium by measuring the efflux of ingested FITC-dextran into the serum. As shown in Fig. 5A, the serum levels of FITC-dextran were significantly higher in the DSS-induced UC mice than those in the control group, indicating that DSS intervention disrupted the intestinal barrier. However, treatment with Em, EmB, and EmB-MO led to a marked reduction in the serum FITC-dextran levels, and the effect was more pronounced in mice treated with EmB or EmB-MO. Intercellular tight junction (TJ) proteins such as Claudins, ZO-1, and Occludin maintain the intestinal barrier integrity. Therefore, we also analyzed the expression levels of Claudin 1, Claudin 4, ZO-1, and Occludin mRNAs and proteins in the colon tissues from the different treatment groups. As shown in Fig. 5B, C, all TJ proteins were significantly downregulated following DSS intervention, which is consistent with previous findings [12]. EmB and EmB-MO restored the levels of TJ proteins, and EmB-MO caused more significant changes than EmB (Fig. 5B, C). Taken together, EmB-MO can effectively restore the gastrointestinal epithelium and repair intestinal barrier function in mice with DSS-induced UC by upregulating the TJ proteins.

### EmB-MO selectively accumulated in inflammatory intestinal tissues

**Fig. 6:**
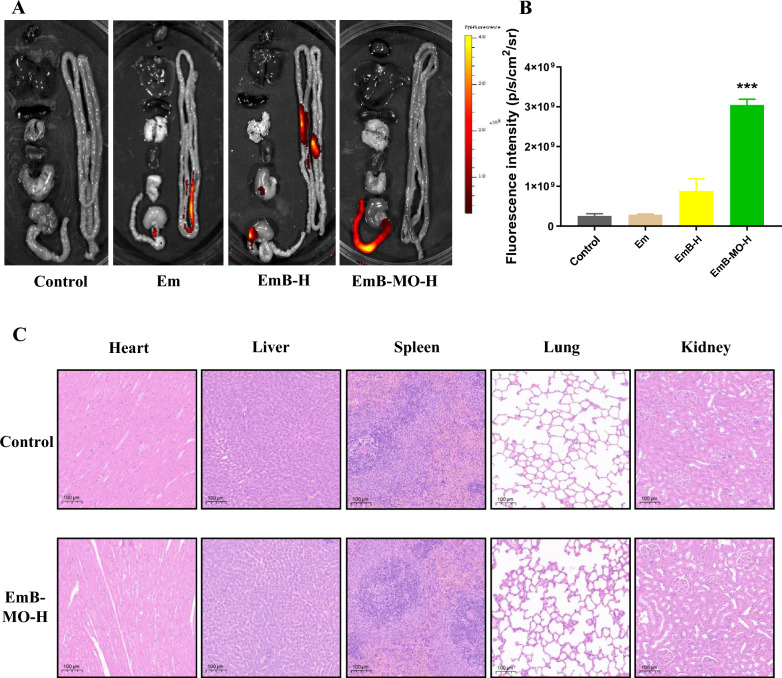
Targeted accumulation of Em, EmB, and EmB-MO in the inflammatory colon. **A** Fluorescence in the heart, liver, spleen, lung, kidney, stomach, small intestine, and colon of normal mice and ulcerative colitis mice 12 h after the oral administration of cy7-Em, cy7-EmB, and cy7-EmB-MO. **B** Fluorescence intensity in each group. **C** Representative micrographs of H&E staining in the heart, liver, spleen, lung, and kidney for the evaluation of drug toxicology at animal level. Data represent the mean ± standard error of the mean (n = 3), ^***^P < 0.001 vs. control group. Em: Emodin, 8 mg/kg/d; EmB-H: Emodin-borate nanoparticles, 6 mg/kg/d; and EmB-MO-H: EmB modified with oligomeric mannitol into microgels, 6 mg/kg/d

The targeted accumulation of the different formulations was assessed in terms of their fluorescence intensities in the colon tissues and other organs. As shown in Fig. 6A, the fluorescence intensity of the inflamed colon in the EmB-MO-H group was apparently higher than the other three groups 12 h after the administration of cy7-labeled Em, EmB, and EmB-MO. Meanwhile, intense fluorescence signals were detected in the small intestine of the Em and EmB-H groups, whereas weak fluorescence signals were detected in the heart, liver, spleen, lung, kidney, stomach, and small intestine of the EmB-MO-H group, which confirmed the selective accumulation of EmB-MO in the inflamed colon tissues. Consistent with the imaging results, the quantification of fluorescence intensities in the colon also indicated that EmB-MO selectively accumulated in the inflamed lesions of the colon without damaging the healthy tissues (Fig. 6B). As EmB-MO preferentially bound to the mannose receptor on the surface of macrophages via the mannitol oligomers [49], the targeted accumulation of EmB-MO in the inflamed colon may mitigate macrophage-driven inflammation. However, the different receptors on the surface of macrophages interact with each other and exert multiple functions [50]. Therefore, the actual mechanism of action of EmB-MO needs further clarification.

Considering the several toxic side effects of Em, such as hepatoxicity, nephrotoxicity, genotoxicity, and developmental toxicity [51], we assessed the toxicity of EmB-MO in healthy tissues. Compared with the control group, no typical pathological changes were observed in the heart, liver, spleen, lung, and kidney of the EmB-MO-H group (Fig. 6C), suggesting that the synthesis of EmB and its modification with oligomeric mannitol into microgels effectively inhibits systemic absorption and the subsequent hepatotoxicity and nephrotoxicity in UC therapy.

## Conclusion

The synthesis of EmB greatly improved the drug solubility of emodin at the administration site. EmB-MO microgels bound to mannose receptors on the surface of intestinal macrophages and significantly alleviated colonic inflammation and intestinal barrier defects in mice with DSS-induced UC, which translated to the mitigation of UC symptoms. Thus, EmB-MO is a promising targeted drug for UC that can avoid drug damage to healthy tissues and overcome the shortcomings of traditional UC drugs. Thus, it warrants further development for prospective clinical applications.

## Data Availability

All data generated or analysed during this study are included in this published article.

## Abbreviations

UC: Ulcerative colitis
Em: Emodin
EmB: Emodin-borate nanoparticles
EmB-MO: EmB modified with oligomeric mannitol into microgels
DSS: Dextran sulfate sodium
MO: Oligomeric mannitol
ELISA: Enzyme linked immunosorbent assay
RT-PCR: Real‑time polymerase chain reaction
H&E staining: Hematoxylin–eosin staining
OCDDS: Oral colon targeted drug delivery system
DAI: Disease activity index
5-ASA: 5-Aminosalicylic acid
MPO: Myeloperoxidase
Lcn-2: Lipoprotein-2
FITC-dextran: Fluorescein isothiocyanate dextran
IVIS: In vivo Imaging system
IBD: Inflammatory bowel disease
TJ proteins: Tight junction proteins
